# Person Re-ID by Fusion of Video Silhouettes and Wearable Signals for Home Monitoring Applications †

**DOI:** 10.3390/s20092576

**Published:** 2020-05-01

**Authors:** Alessandro Masullo, Tilo Burghardt, Dima Damen, Toby Perrett, Majid Mirmehdi

**Affiliations:** Department of Computer Science, University of Bristol, Bristol BS8 1UB, UK; tb2935@bristol.ac.uk (T.B.); Dima.Damen@bristol.ac.uk (D.D.); toby.perrett@bristol.ac.uk (T.P.); M.Mirmehdi@bristol.ac.uk (M.M.)

**Keywords:** sensor fusion, digital health, silhouettes, accelerometer, ambient assisted living

## Abstract

The use of visual sensors for monitoring people in their living environments is critical in processing more accurate health measurements, but their use is undermined by the issue of privacy. Silhouettes, generated from RGB video, can help towards alleviating the issue of privacy to some considerable degree. However, the use of silhouettes would make it rather complex to discriminate between different subjects, preventing a subject-tailored analysis of the data within a free-living, multi-occupancy home. This limitation can be overcome with a strategic fusion of sensors that involves wearable accelerometer devices, which can be used in conjunction with the silhouette video data, to match video clips to a specific patient being monitored. The proposed method simultaneously solves the problem of Person ReID using silhouettes and enables home monitoring systems to employ sensor fusion techniques for data analysis. We develop a multimodal deep-learning detection framework that maps short video clips and accelerations into a latent space where the Euclidean distance can be measured to match video and acceleration streams. We train our method on the SPHERE Calorie Dataset, for which we show an average area under the ROC curve of 76.3% and an assignment accuracy of 77.4%. In addition, we propose a novel triplet loss for which we demonstrate improving performances and convergence speed.

## 1. Introduction

Ambient Assisted Living (AAL) is now a well-established area of research, fuelled by the continuously ageing and longer life expectancy of the population [1]. The majority of video monitoring applications for AAL make use of RGB images to provide clinically relevant measurements [2]. While the presence of monitoring CCTV (Closed-Circuit Television) cameras is nowadays accepted in public spaces, such as shops and city centres, due to privacy reasons, people are often reluctant to install them in their own homes for any purpose other than security, for example, even for constant health monitoring [3,4,5]. Silhouettes constitute an alternative form of data for video monitoring in privacy-sensitive environments [6]. Due to their light weight representation, silhouettes are often the preferred form of data in Internet of Things (IoT) and AAL applications [7]. In our previous works, we demonstrated that silhouettes can be reliably employed for long-term home monitoring applications to measure important health-related parameters, for example, measurement of calorie expenditure [8] and the speed of transition from sitting to standing or standing to sitting (StS) [9], which are proxy measurements for sedentary behaviour, musculoskeletal illnesses, fall history and many other health-related conditions. Previous work in the field also showed that silhouettes can be successfully employed for fall detection [10] and abnormal gait analysis [11]. The recent work from Colantonio et al. [12] highlighted some of the challenges that are still open in AAL from a Computer Vision perspective, including the assessment of reliability of these measurements for clinical purposes, the robustness of the operating conditions in real-life settings and the final user acceptance of the monitoring system, which we will address through this paper.

Under the auspices of the SPHERE project [13] (a Sensor Platform for HEalthcare in a Residential Environment), we recorded data from voluntary participants in 52 real-world homes, including silhouettes, body accelerations and a variety of different environmental sensors’ data. The latter are outside the scope of this present work and not used here. The analysis of these silhouettes allows monitoring the health of the participants while respecting their privacy; however, silhouette-based measurements are limited in that they cannot be assigned to a specific individual in the house. Due to their anonymous and often noisy nature, silhouettes hinder the discrimination of different individuals, preventing a subject-tailored analysis of the data. This problem is of critical importance for scenarios of home monitoring, particularly in long-term observations. For example, in the HEmiSPHERE project [14], patients undergoing hip and knee replacement spend their rehabilitation period at home while being monitored with the SPHERE sensors. It is essential to be able to automatically discriminate between the silhouettes of the patient to be monitored and the rest of the household or occasional guests such that clinicians can investigate the recovery trends of their patient only, while also respecting the privacy of all those within view.

The solution we propose is to take advantage of wrist-worn accelerometers, for which the measurements can be unequivocally assigned to the person wearing it. Matching the motion from the video silhouettes with the motion from the accelerometers enables us to assign each video measurement to a specific individual. Thanks to this approach, not only can we reliably monitor each participant in the house but we can also enable a sensor fusion approach to improve the quality of the silhouette-based measurements, overcoming the limitations of both wearables and videos. While matching video and acceleration streams has already been attempted in the past, previous works only focused on the use of RGB images and long observation times (i.e., >1 min). Moreover, existing methodologies require all subjects appearing in the video to be carrying an accelerometer, which is not suitable for real world monitoring applications. Patients may have guests, and they cannot be required to wear accelerometers at all times.

**Figure 1:**
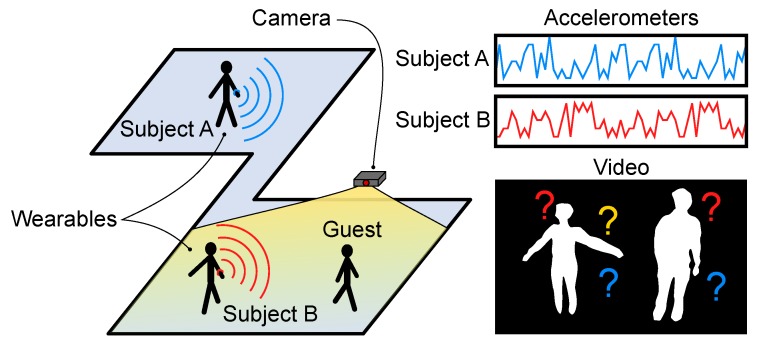
Description of a typical real-life scenario for home monitoring. Two subjects (A and B) are wearing an accelerometer, but only one of them appears within camera view, together with a guest. Our aim is to understand which of the two monitored targets appears in the video silhouette frames.

In this paper, we extend our previous work [15] on the Video-Acceleration Matching problem that sets new state-of-the-art results in privacy-sensitive Home Monitoring applications. We consider real-life monitoring scenarios and we tackle the rather complex case where only the monitored participants wear an accelerometer whilst being visually recorded amongst other persons, as Figure 1 illustrates. Moreover, our pioneering solution to the Video-Acceleration matching problem can operate on even short (≈3 s) video snippets, so that quick and clinically relevant movements (e.g., Sit-to-Stand [9]) can be associated to a specific individual in spite of the length of the event. We propose a novel multimodal deep-learning detection framework that maps video silhouettes and accelerations into a latent space where the Euclidean distance can be measured to match video and acceleration streams. Further, we propose a novel loss function that may be used as an alternative to triplet loss for dual stream networks. We present results for video-acceleration matching on the challenging SPHERE-Calorie dataset [16,17].

## 2. Related Work

The main objective of our work is to identify people from a video stream of silhouettes. We group the related literature for re-identification into three broad categories: methods that operate on a persons appearance in single images, methods that utilise videos, and methods that exploit the fusion of two different modalities (i.e., video-acceleration matching). We also review some of the works related to Audio-Video Synchronisation, because of the very close similarity of this problem with the Video-Acceleration Matching.

### 2.1. ReID from Images

**Appearance based**—Identifying people from their silhouettes can be approached as a re-identification (ReID) problem [18,19,20]. The vast majority of the literature on person ReID makes use of RGB images, as detailed in the review from Bedagkar-Gala et al. [21] and the more recent deep-learning review from Wu et al. [22]. Deep-learning algorithms on RGB images constitute the state-of-the-art in person ReID; however, as addressed in [22], the majority of them are mainly focused on short-term scenarios. Person ReID for home monitoring includes very demanding challenges, such as strong change in appearance (i.e., clothes), appearance impaired scenarios, etc., that need to be specifically addressed. While some recent works have tried to tackle these issues [23,24], the use of RGB images remains unsuitable for home monitoring due to ethical reasons [5]. In fact, while ReID features could potentially be computed with RGB images before they are discarded, the use of deep-learning algorithms requires a large computational power, which is often not available on the small IoT devices used for monitoring. Outsourcing this computation to external servers exposes serious dangers for the privacy of the monitored subjects and strict ethical restrictions prevent such a solution.

**Motion based**— To deal with appearance impaired scenarios and solve the problem of long-term ReID, several works make use of motion to re-identify people, in particular (temporal) gait [25]. Features like the transitional characteristic of gait [26], motion patterns on tracklets [27] and deep learning motion descriptors [28] have been successfully employed to re-identify people while walking, either using silhouettes or RGB images. The hypothesis behind these works is that every person walks in a different way, exhibiting patterns that can be exploited to identify them. While this hypothesis is valid for ReID of pedestrians, it does not apply to indoor scenarios, where smaller environments do not allow for full development of gait sequences. Moreover, as we showed in [9], patients undergoing physically-impairing surgery will show drastic changes in their mobility. Such changes completely violate the main hypothesis of motion-based ReID algorithms, rendering them inapplicable to long-term clinical monitoring.

### 2.2. Audio-Video Synchronisation

The Audio-Video Synchronisation task carries similar challenges to the Video-Acceleration Matching problem, and many works can be found on this topic, such as [29,30,31]. In particular, Active Speaker Detection is the problem of identifying which of the people in a video an utterance can be attributed to at a given time, and correspondingly, the ReID from silhouettes domain requires to establish which accelerometer can be attributed to which person. Chung et al. [32] proposed a two-stream architecture that learns a joint embedding between the sound and the motion of the lips from a video. The network is trained using sequences of frames for the video and Mel-frequency cepstral coefficients (MFCC) for the corresponding audio. Synchronisation between audio and video was also used by Korbar et al. [33] as a form of self-supervised training, exploited to learn useful features for secondary tasks, like classification of videos and sounds.

### 2.3. Matching Video and Acceleration

**Matching trajectories**— The idea of matching video features with accelerations to identify subjects in front of a camera has already been explored in the past, and one of the earliest approaches matches trajectories derived from video and acceleration streams [34]. They suggested a probabilistic approach that maximises the likelihood that subject locations extracted from the cameras correspond with the locations produced by the inertial sensors. Jiang et al. [35] used Histogram of Oriented Gradient (HOG) descriptors and a Support Vector Machine (SVM) to generate tracks from RGB images of pedestrians, and then compared them with dead-reckoning paths integrated from Inertial Measuring Units (IMU) carried by the recorded subjects. Henschel et al. [36] adopted a graph labelling formulation that integrates body worn IMUs and trajectories extracted from a video camera to solve the Video Inertial Multiple People Tracking problem. While the approach of comparing trajectories to solve the Video-Acceleration Matching problem works well for outdoor scenarios, it is not suitable for indoor free-living monitoring. In fact, many of the typical indoor activities of daily living do not necessarily require the transition from two different places (e.g., eating, ironing, washing dishes, watching TV, and so on). Moreover, these methods are completely reliant on the performances of the trackers, and IMU based trajectories are particularly affected by a strong bias that accumulates over time due to the double integration involved in the computations [37].

**Acceleration from video**—A different approach to tackle the Video-Acceleration Matching problem is to estimate accelerations from the video stream. In Shigeta et al. [38], video frames are segmented based on motion and the centroid of each detected area is used to estimate the acceleration vector. Rofouei et al. [39] follow a similar approach using the position of skeleton joints to estimate the acceleration, while Wilson et al. [40] estimate the acceleration field using dense optical flows from an infrared camera, which are converted into 3D flows using depth information. All these methods are limited to cases where the wearable device is in the line of sight to the camera, which is not a reliable proposition. In [41,42], Cabrera-Quiros et al. tackle the case of crowd-mingling events that include dozens of participants, recorded by cameras, accelerometers and proximity sensors. They estimate acceleration from the video optical flow and use the measurements from proximity sensors to cluster neighbouring people and hierarchically associate them to wearables. A strong limitation of this approach is that every person in the room needs to be carrying the proximity device for the hierarchical method to work. Moreover, their method requires several minutes of recording before being able to reliably match video and acceleration streams, which may be unsuitable for cases where the subjects frequently move in-between rooms. In spite of these limitations, the work from Cabrera-Quiros et al. is the state-of-the-art in Video-Acceleration Matching. It is the closest work to our proposed methodology, and we shall apply it for our comparative evaluation.

## 3. Materials and Methods

Before matching video sequences with accelerations, the video stream must be processed to detect different subjects appearing in the frame. In our work, we use the person detector and tracker from OpenNI [43], which provides bounding boxes and tracking information. Similarly to the works in Active Speaker Detection, we developed our framework to match short video/acceleration clips (≈3 s). The reason behind this choice is that we are interested in identifying subjects while performing short, clinically relevant movements. Shorter clips also helps to minimise possible errors of the trackers, for example, exchanging bounding boxes of different subjects.

### 3.1. Video-Acceleration Matching

A typical installation of a real-life home in the SPHERE project [6] provides for a camera in each communal room and corridor, and an accelerometer for each participant. Guests can visit the monitored house at any time but will not carry an accelerometer. All the video and accelerometer sensors are synchronised via their time-stamps.

Let us consider a set of *K* silhouette video clips $V=\{v_{1},\ldots ,v_{K}\}$ portraying one person at a time (i.e., the sequence of frames cropped around the bounding boxes) while wearing the wristband. Time-synchronised acceleration samples from the wristband are also recorded and grouped into consecutive sequences $A_{p}=\left\{a_{1},\ldots ,a_{K}\right\}$. The accelerations $A_{p}$ constitute a positive match for the videos *V* by construction. We can define a set of non-matching accelerations $A_{n}$ by selecting for each $V_{i}$ the acceleration from a different monitored subject (details on different types of negatives will be discussed in Section 3.3). The objective of the video-acceleration matching is to find two optimal encoding functions f(·) and g(·), so that the Euclidean distance *d* is minimised for $d\{f\left(V\right),g\left(A_{p}\right)\}$ and maximised for $d\{f\left(V\right),g\left(A_{n}\right)\}$. The functions *f* and *g* are two CNNs that take as input of the video clip and the raw accelerations respectively, and produce for output feature vectors. During testing, the matching between a generic video stream and a specific accelerometer can be verified by comparing the Euclidean distance of the two encoded streams with a threshold, the optimal value of which can be derived from the Receiver Operating Characteristic (ROC) curve described in Section 4.1.

### 3.2. Loss Function

One potential way to address the problem of video-acceleration matching is to reformulate it as a classification problem. Given the videos *V* and the accelerations $A_{p}$ and $A_{n}$, we can build the pairs $\left(V,A_{p};1\right)$ and $\left(V,A_{n};0\right)$ for the classes “matching” and “non-matching”. With this setting, standard cross-entropy can be used to train the video and acceleration encoders f(·) and g(·). However, it has been shown in [33] that for audio and video matching, the binary classification task constructed in this way is difficult to train and we therefore discarded it.

A valid alternative to the cross-entropy for binary classification is the triplet loss, that was first proposed to train Siamese Networks for face recognition [44]. A triplet is defined as a set of three elements comprising an anchor, a positive match and a negative match. Here, we use the video as anchor, and a matching and non-matching sequence of accelerations for the positive and the negative match respectively,

(1) $$\left(\mathrm{anchor},\mathrm{positive},\mathrm{negative}\right)\equiv \left(V,A_{p},A_{n}\right).$$

**Figure 2:**
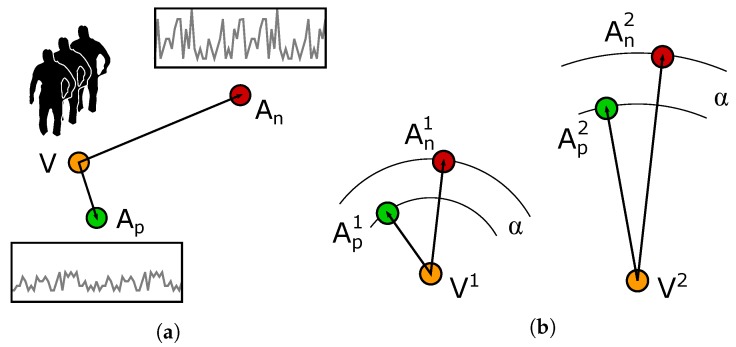
(**a**) Example of triplet constituted by an anchor video of silhouettes and two acceleration sequences for positive and negative matches. (**b**) Possible problem occurring while training with the standard triplet loss and a fixed margin *α*.

With this definition of triplet, the loss is defined as:

(2) $$L_{\mathrm{triplet}}=max\left\{{\left|f\left(V\right)-g\left(A_{p}\right)\right|}^{2}-{\left|f\left(V\right)-g\left(A_{n}\right)\right|}^{2}+\alpha ,0\right\}$$

where α is a constant, empirically set to 0.2. The behaviour of the triplet loss is described in Figure 2a: by minimising the quantity described in Equation (2), the pairs of $\left(V,A_{p}\right)$ are pulled together, while $\left(V,A_{n}\right)$ are pushed apart, to a distance greater than α.

In addition to the standard triplet loss, we also experimented using alternative formulations that take advantage of the triplets. One of the problems we experienced with the standard triplet loss is that it does not guarantee that a single threshold can be used to discriminate between matching and not-matching pairs. In fact, the objective of the triplet loss is to separate the $\left(V,A_{p}\right)$ pair from the $\left(V,A_{n}\right)$ pair, no matter what the intra-pair distances are. For example, given two triplets $T^{1}\equiv \left(V^{1},A_{p}^{1},A_{n}^{1}\right)$ and $T^{2}\equiv \left(V^{2},A_{p}^{2},A_{n}^{2}\right)$ as described in Figure 2b, optimising for the standard triplet loss ensures that:

(3) $$d\left\{f\left(V^{1}\right),g\left(A_{n}^{1}\right)\right\}-d\left\{f\left(V^{1}\right),g\left(A_{p}^{1}\right)\right\}>\alpha$$

and

(4) $$d\left\{f\left(V^{2}\right),g\left(A_{n}^{2}\right)\right\}-d\left\{f\left(V^{2}\right),g\left(A_{p}^{2}\right)\right\}>\alpha .$$

However, it is entirely possible that the distances are such that $d\left\{f\left(V^{2}\right),g\left(A_{p}^{2}\right)\right\}\gg d\left\{f\left(V^{1}\right),g\left(A_{n}^{1}\right)\right\}$. As it will be shown later, this behaviour is very common for some training strategies and renders the model inoperative, since no single threshold can be used to discriminate between matching and non-matching sequences.

The objective of the training must therefore be such that the model can be used with a single universal threshold. The limitation of the standard triplet loss is that it becomes identically zero once the distances in Equation (2) are greater than α. To overcome this limitation, we propose a new loss function, Reciprocal Triplet Loss ($L_{\mathrm{RTL}}$), which does not involve any distance margin α and continuously optimises the distances between anchor, positive and negative match:

(5) $$L_{\mathrm{RTL}}={\left|f\left(V\right)-g\left(A_{p}\right)\right|}^{2}+\frac{1}{{\left|f\left(V\right)-g\left(A_{n}\right)\right|}^{2}}$$

As it will be shown in the experiments, the use of the RTL function helps in improving the performance of our model and enables it to be operable more robustly with a single universal threshold.

### 3.3. Negative Samples

When the standard triplet loss is used to train a deep learning model, the samples constituting each triplet must be cleverly selected in a way that they can actively contribute to improving the model. In fact, if the distance between the video anchor and the accelerations from Equation (2) is greater than α, the triplet will have zero loss and it will not contribute to the training. In the original paper on the triplet loss [44], hard mining of triplets was considered as a crucial step to deal with this problem. In our case, the triplets are constrained by the problem of matching videos with accelerometers, and the anchor-positive pair must be a video clip with the matching acceleration sequence. However, the choice of the non-matching acceleration can vary substantially and it has a strong effect on the outcome of the training process.

**Figure 3:**
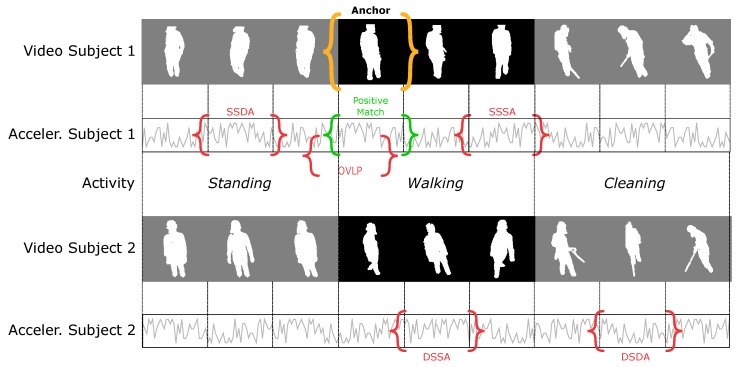
Description of the different possibilities for the negative samples in the triplet. The anchor is the video clip marked in orange, while the positive match is marked in green. A single example of each different negative sample is marked in red.

Let us consider an example where a group of *N* subjects $\left({\mathrm{Sub}}_{1},\ldots ,{\mathrm{Sub}}_{N}\right)$ is performing a set of activities (*standing*, *walking*, *cleaning*, …). Given an anchor video portraying a subject doing a specific activity, as depicted in Figure 3, a non-matching acceleration can be selected from a different subject doing a different activity (DSDA) or doing the same activity (DSSA), or it could be from the same subject doing the same activity (SSSA) or a different activity (SSDA). The possible combinations of negative samples are summarised in Table 1 for clarity.

The objective of this work is to train a model that learns the matching between video and acceleration streams. However, if negative samples are only drawn from a different subject doing a different activity (DSDA), the video-acceleration matching problem degenerates into a simple activity or identity classifier. Let us consider, for example, a triplet where the anchor is the video of ${\mathrm{Sub}}_{1}$ while “walking”. The positive match will be the acceleration of ${\mathrm{Sub}}_{1}$ while “walking”, whereas a DSDA negative could be ${\mathrm{Sub}}_{2}$ doing “cleaning”, as depicted in Figure 3:

(6) $$\left(V,A_{p},A_{n}\right)\equiv \left(\left\{\mathrm{Sub}\;1;Walking\right\},\left\{\mathrm{Sub}\;1;Walking\right\},\left\{\mathrm{Sub}\;2;Cleaning\right\}\right).$$

Since the non-matching acceleration $A_{n}$ will always be from a different subject doing a different activity, the neural network will try to learn either the identity of the subjects or the activity being performed through the encoding functions f(·) and g(·). Equivalently, training only with DSSA negatives reduces to an activity-agnostic identity classifier, while training with SSDA negatives leads the classifier to only learn activities. A model trained exclusively on DSDA, DSSA or SSDA negatives will not learn anything about the actual correlation between the video and the accelerations, but it will merely compare the action or identity predicted from the video with the one predicted from the accelerations. This type of model is therefore expected to fail when tested on unseen subjects or activities.

To overcome this limitation and truly associate visual and acceleration features in the temporal domain, a non-matching acceleration can be selected from the same subject while performing the same activity (SSSA). We call this type of negative “hard-negative” (in contrast to the “easy-negatives” DSDA, DSSA and SSDA), since a simple activity or subject classifier is unable to solve this problem and it requires the network to encode information about the actual correlation between video and accelerations. Similarly to Korbar et al. [33], we also consider a further type of negative sample constituted by an acceleration that is out-of-synchronisation with the video but it is still overlapping with it, as presented in Figure 3. The out-of-synchronisation negative will be very similar in shape to the synchronised positive match; we call this type of negative overlapping (OVLP), and we refer to is as “very hard-negative”. It is important to clarify that amongst all the negative types tested, those from the “Same Subject” or “Overlapping” category are only used for training purposes, since the same subject cannot really appear in multiple locations of the same video clip.

In this work, we tested a variety of training strategies that include different combinations of easy, hard and very-hard negatives, as described in Table 2. From an inference point of view, the same subject cannot appear in multiple locations at the same time, therefore the validation data only includes negative types of DSDA and DSSA, while the SSSA and SSDA negative types are only used for training.

The data used in this study (described in detail in Section 3.7) was split into training and testing based on subject identities, so that the subjects used for testing were never seen during training. Regarding the choice negative samples, a 50% balance between DSDA and DSSA was chosen and was kept constant across all the experiments.

### 3.4. Data Preprocessing

Silhouettes from each subject detected in the scene are cropped around the bounding boxes and resized to a constant value of 100 pixels (keeping the original aspect-ratio). The video sequence of each subject is then truncated into short clips of 100 frames (≈3 s) each. In order to avoid any loss of information from the cropping process, bounding box coordinates are also fed into our video encoder network together with the silhouettes. The logic behind this is that the human body can be seen as a deformable body that can either translate or change its shape, and bounding boxes will better capture large rigid displacements (e.g., walking) while the cropped silhouettes will address smaller changes within the body shape (e.g., wiping a surface).

The accelerometer data comprises a 3-channel vector, i.e., the IMU measurements in *x*, *y* and *z*. Typically, machine learning algorithms for audio analysis make use of some transformation of the audio signal in the frequency domain, for example, using Perceptual Linear Predictive coefficients (PLPs) [45] or variations of the MFCC [32,46,47]. However, since the accelerometer signal is sampled at a frequency that is several orders of magnitude lower than audio (typically around 50 Hz for IMU [48] and 32–48 kHz for audio [49]), we feed the raw amplitude of the accelerometers into the network, leveraged by our previous work [8] where we observed that the direct convolution of acceleration amplitudes yielded satisfactory results. Due to the variability of the sampling rate of the accelerometers used in our experiments, the only pre-processing we perform on the acceleration stream is sub-sampling of the data to match the video frame rate (while sub-sampling in our experiment was dictated by the minimum sampling rate of the accelerometer devices, our method does not require the video sampling rate to match the accelerometer and it can be applied to mismatching rates by simply adjusting the input size of the network). Conceptually, this leaves data transforms to be a responsibility of the network itself.

### 3.5. Network Architecture

The most important element of our algorithm is the function of the two encoders f(·) and g(·), represented by different CNNs, that process the video and acceleration streams independently to produce the feature vectors. In particular, the video encoder f(·) is the sum of the silhouettes encoder $f_{\mathrm{sil}}\left(\cdot \right)$ and the bounding box encoder $f_{\mathrm{bb}}\left(\cdot \right)$. Our encoders $f_{\mathrm{sil}}\left(\cdot \right)$, $f_{\mathrm{bb}}\left(\cdot \right)$ and g(·) then constitute a three-stream architecture that is able to take video and acceleration data as input and produce the distance between the two in the latent space as output.

**Figure 4:**
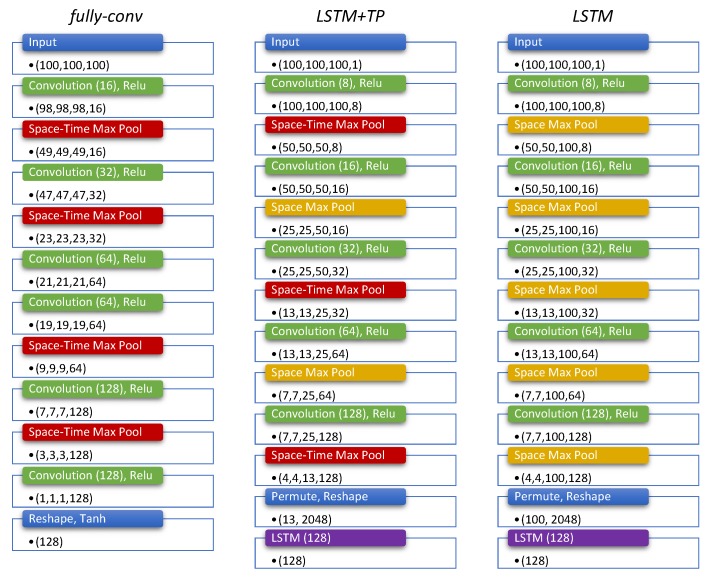
Architecture of the video branch $f_{\mathrm{sil}}\left(\cdot \right)$ for the three networks tested in this work. The other branches $f_{\mathrm{bb}}\left(\cdot \right)$ and g(·) present the same architecture with 3D operators replaced by the 1D counterpart.

We implemented 3 different architectures, as illustrated in Figure 4, and tested their performances under different conditions to find the best configuration.

**Fully Convolutional(*fully-conv*)—** The idea behind deploying this architecture is to reduce the input size using a sequence of convolution and max-pooling operations while simultaneously increasing the number of features being produced. For the video branch only, both convolution and max-pooling operations are performed with a 3D kernel to extract spatio-temporal information, while 1D kernels are used for the branches processing bounding boxes and accelerations.

**LSTM with temporal pooling(*LSTM+TP*)**— This architecture is similar to the fully convolutional network, but it uses fewer max-pooling layers in time, so that a temporal feature vector is produced by the convolutional stack. This temporal feature vector is fed into a Long Short-Term Memory (LSTM) layer that produces the final embedding.

**LSTM without temporal pooling(*LSTM*)**— Inspired by [45], we developed an architecture that does not involve any temporal pooling at all. The expectation here is that fine temporal information is important to learn the correlations between video and acceleration streams and helps to provide better results with the matching task. As in the previous case, the final temporal feature vector is fed into an LSTM layer to produce the final embedding vector.

All the architectures tested used a kernel size of 3, spatial dropout after each convolutional layer and activation ReLu. Only the *fully-conv* architecture uses a final activation using *tanh* after the last convolution, while the remaining networks use the standard LSTM internal activation functions.

### 3.6. Baseline Method

In order to show the advantages of our method, we implemented some algorithms from the literature to use for baseline comparison. The first work is the state-of-the-art method for matching video and wearable acceleration streams by Cabrera-Quiros et al. [41], where they estimate acceleration data from the video stream using dense optical flow and then compare it with the actual acceleration stream. The wearable devices adopted in their experiment also included an embedded proximity sensor that they used to cluster neighbouring devices. Since the target of our study is matching video and acceleration streams without any further sensor input, we implemented their algorithm without the hierarchical approach for the Hungarian method.

In addition to Cabrera-Quiros et al., we also implemented a method inspired by Shigeta et al. [38]. In their work, accelerations are estimated using the centroid of each bounding box detected in the video stream and are compared with a low-pass filtered version of the acceleration stream. While implementing this work, our experiments showed that better results were achieved using a high-pass filtered version of the acceleration. Moreover, Shigeta et al. use normalised cross-correlation to compare the video and accelerometer signal because their target is streams that are temporally not synchronised. Since we are dealing with a case where the video and acceleration streams are always synchronised, we compared the signals using Euclidean distance, as per our work.

### 3.7. Dataset

**Figure 5:**
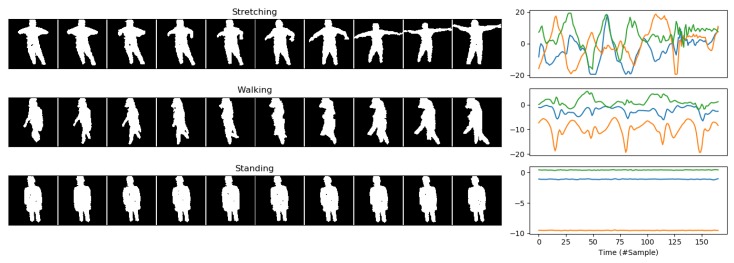
Samples of video and acceleration signals from the Calorie dataset for three specific activities.

Our dataset is a modified version of the SPHERE-Calorie dataset [16], which includes RGB-D images, bounding boxes, accelerations and calorie expenditure measures obtained from a Calorimeter, from 10 different individuals doing a set of 11 activities in two different sessions. In this work, we discarded the calorie data and converted the RGB-D images into silhouettes. Silhouettes were generated by processing the RGB images with OpenPose [50] to extract the skeleton joints for each frame of the dataset and then running GrabCut on the depth images using a mask initialised with detected skeletons. Samples for the silhouettes and accelerations in the dataset are shown in Figure 5. The dataset includes 11 different activities, from which we kept actions that involve movement (i.e., walking, wiping a surface, vacuuming, sweeping, exercising, stretching, cleaning). The dataset includes more than 2.5 million individual silhouettes, which were used to generate ≈50,000 video clips (and matching acceleration sequences). To the best of the Authors’ knowledge, the SPHERE-Calorie dataset is the only large dataset including RGB-D and acceleration data from wearable devices that is suitable for the Video-Acceleration Matching problem using silhouettes (while the Authors are aware of the existence of the MatchNMingle dataset [51], the combination of high view angle (top view) and low focal lens used to record the RGB data make it very difficult to generate silhouettes).

The data from the SPHERE-Calorie dataset was recorded one subject at a time, which enabled us to automatically pair the correct matches between videos and wearables. To simulate the presence of multiple people in each room, we followed the widely adopted strategy of virtual streams [34,41] whereby the video and acceleration streams were split into smaller intervals and treated as if they were occurring at the same time. While this approach might be limiting in that subjects never interact with each other, it allows us to push the number of subjects present in a frame beyond the actual capacity of a room, assessing the limits of our method. The split between training and testing was performed based on the subject identities: subjects 1 to 7 for training the algorithm and subjects 8 to 10 for testing. This split ensured that the network could not exploit any visual appearance cues to identify people and forced it to learn the actual correlation between video and acceleration streams.

### 3.8. Implementation Details

All the networks tested were trained end-to-end using the silhouette video and acceleration streams in input and the triplet of distances over the embedding in output. The code was implemented using Keras and Tensorflow in Python (the code will be available on GitHub at: https://github.com/ale152/video-accelerometer-matching). Training was performed using the optimiser Adam [52] with a learning rate of $10^{-4}$ and a batch size of 16. We monitored the area under the ROC curve (auROC) after each epoch (as later detailed in Section 4.1) using the validation data and we stopped training when none of the auROC scores improved for more than 50 epochs. In order to improve performances on the validation data, we implemented some data augmentation strategies. Both the streams of video and acceleration data were truncated to short clips of ≈3 s each using 95% overlap. In addition to that, data augmentation for the video silhouettes was implemented by randomly flipping (horizontally), dilating (up to 5 pixels), eroding (up to 5 pixels) and corrupting with salt-and-pepper noise (3%). This strategy, combined with a spatial dropout employed after each convolutional layer, was designed to reduce overfitting of the models on the training data. When OVLP negatives are used, the negative clip is randomly selected to be from −10 to +10 frames out-of-sync with the positive match.

## 4. Experiments and Results

We present a series of experiments and ablation tests that are targeted at understanding the advantages and performances of our novel method compared to the state-of-the-art. We tested all possible combinations of models (*fully-conv*, *LSTM+TP*, *LSTM*) and training strategies (*Easy*, *Easy/Hard*, *Hard*, *Hard/VeryH*, *VeryH*, *All*) for both the Standard Triplet Loss and our proposed Reciprocal Triplet Loss.

### 4.1. Area under the ROC Curve

We first evaluate our method on the matching verification task: given a video clip $V_{i}$ and an acceleration $A_{j}$, the Euclidean distance between the two embedding $f(V_{i})$ and $g(A_{j})$ is compared with a threshold τ to determine the outcome of “matching” or “not matching”. While the true matching pairs *P* are unequivocally defined by the correct pairs of video and acceleration, the true non-matching *Q* can be any of the possibilities (the reader is reminded that a negative of the “Same Subject” type can never occur in reality, since the same person cannot appear simultaneously in multiple locations; however, we report results for this type of negative because it is useful for our discussion to understand specific behaviours of the models trained) described in Table 1, resulting in a different score for each negative type. We define the correct true positive matches TP, as a function of the threshold τ, such that:

(7) $$TP\left(\tau \right)=\left\{\left(V_{i},A_{j}\right)|f{\left(V_{i}\right)}^{2}-g{\left(A_{j}\right)}^{2}<\tau ,\left(V_{i},A_{j}\right)\in P\right\}\;,$$

and the false positive matches FP as:

(8) $$FP\left(\tau \right)=\left\{\left(V_{i},A_{j}\right)|f{\left(V_{i}\right)}^{2}-g{\left(A_{j}\right)}^{2}<\tau ,\left(V_{i},A_{j}\right)\in Q\right\}\;,$$

where *P* and *Q* are the sets of all positives and all negatives, respectively. By varying the threshold τ, we can plot the true positive rate TPR against the false positive rate FPR, defined as:

(9) $$TPR=\frac{TP}{P}\;\;\mathrm{and}\;\;FPR=\frac{FP}{Q}\;,$$

resulting in a ROC curve. The auROC curves tested with each training strategy (Section 3.3) and the average across negative types (AVG) is presented in Table 3, Table 4 and Table 5.

**Figure 6:**
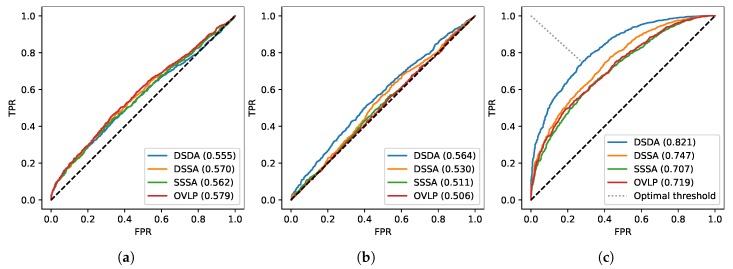
ROC curves for (**a**) Shigeta et al. [38], (**b**) Cabrera-Quiros et al. [41] and (**c**) our proposed algorithm. Optimal threshold for our proposed method is also highlighted in the figure.

A comparison between the three tested architectures reveals that the best performances are obtained by the *fully-conv* model when trained using our novel RTL, with an AVG auROC of 76.3%. The full ROC curves for this particular model are presented in Figure 6c, together with the ROC curves for the baseline method of Shigeta et al. [38] in Figure 6a and Cabrera-Quiros et al. [42] in Figure 6b, which only manage to achieve AVG auROCs of 55.6% and 52.1%, respectively. In terms of best AVG auROC, the best model was trained on a combination of Easy and Hard negatives; while adopting harder negatives samples during training may improve the performances for SSSA auROC, the degradation over other scores leads to a lower AVG auROC. As already expected from the discussion in Section 3.3, the use of Easy negatives exclusively leads to the worst results in the majority of the experiments performed.

If we only consider models trained with the STL, the best one is *LSTM+TP*, trained with a combination of Hard and Very-Hard negatives. This model presents an AVG auROC of 61.7%, which is almost 15% lower than the best model trained with our novel RTL, confirming the advantages of our novel loss function. In addition to that, we also experienced a much faster training when using our proposed RTL, that reached maximum performances in fewer iterations when compared to the STL. Comparing the LSTM models in Table 4 and Table 5, we notice that the absence of temporal pooling with the LSTM layer does improve performances, as predicted in Section 3.5. This improvement is likely related to the more granular temporal information that is fed into the LSTM layer, that better captures the correlation between the video and acceleration streams. However, with auROC values of 71.1% for *LSTM* and 61.7% for *LSTM+TP*, these two models remain inferior to *fully-conv*. Moreover, because of the missing temporal pooling, *LSTM* requires 50% longer training time with respect to *fully-conv* and *LSTM+TP*.

A comparison between the STL and RTL functions across all the models and negative strategies tested shows a clear superiority of our novel loss function in terms of auROC. Although 17 of our 18 experiments support the superiority of RTL over STL, we stress that these results are only valid in the context of the Video-Acceleration Matching problem. While the implementation of the RTL to other Computer Vision problems is straightforward, the performance analysis of our novel loss function for general application is out of the scope of this article.

### 4.2. Temporal Results

**Figure 7:**
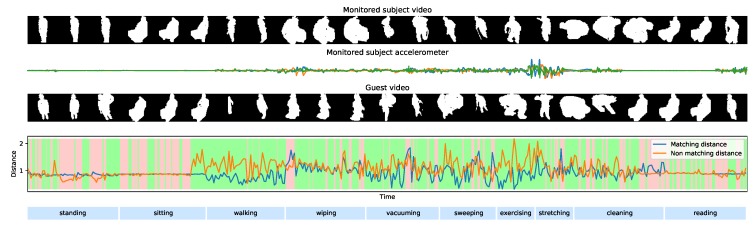
Temporal results for our best model showing the distance between an acceleration sequence and its matching video and the video sequence of a potential guest.

Temporal results for our algorithm are presented in Figure 7 for two example subjects (Subject 9 and Subject 10) from the testing data. We illustrate the situation where both subjects appear in front of the camera but only one of them is wearing a wearable, the other being a guest. The objective is to find which short video clip from each sequence matches the monitored accelerometer. The experiment is even more challenging, since both subjects are simultaneously doing the same sequence of activities. We encoded both the video and acceleration sequences using the f(·) and g(·) deep encoders from the best model we found and we evaluated the Euclidean distances between the two pairs of features:

(10) $$d_{\mathrm{Matching}}=\sqrt{\sum_{i=1}^{N}{\left[f\left(V_{9}\right)-g\left(A_{9}\right)\right]}^{2}}$$

and

(11) $$d_{\mathrm{Non}-\mathrm{matching}}=\sqrt{\sum_{i=1}^{N}{\left[f\left(V_{9}\right)-g\left(A_{10}\right)\right]}^{2}}.$$

The results show the detailed temporal performances for the best *fully-conv* model from Table 3. A very different behaviour can be seen between activities that involve movement (i.e., walking, exercising) and those that do not (i.e., sitting, reading). In fact, active movements involve a variety of gestures that produce a strong motion signature which can be exploited to match video and accelerations. On the other hand, the output signal of the accelerometers while resting is almost identically nil, no matter which person is wearing it, hindering the ability to match different accelerations to different video streams.

### 4.3. Performances Varying the Number of People

In this subsection, we set out to study the performance of our matching algorithm when varying the number of subjects, focusing on the effect of multiple guests. We define the number of people appearing in front of the camera $N_{\mathrm{vid}}$ (not necessarily wearing an accelerometer) and the number of people carrying a wearable $N_{\mathrm{acc}}$ (not necessarily appearing in front of the camera). By defining $N_{\mathrm{sync}}$ to denote the number of subjects appearing in front of the camera while carrying the accelerometer, we can compute the number of guests $N_{\mathrm{guest}}$ (people appearing within camera view while not wearing an accelerometer) as

(12) $$N_{\mathrm{guest}}=N_{\mathrm{vid}}-N_{\mathrm{sync}}\;.$$

To study all the possible circumstances that can apply to a regular house, we designed three different experiments. In the first, we fix $N_{\mathrm{sync}}=1$ while we vary $N_{\mathrm{acc}}$ from 2 to 10. This experiment reflects a condition where one single person is wearing the accelerometer in front of the camera, while other monitored participants are in different rooms or simply not in the field of view of the video system. We compute the distance (in the learnt embedded space) between the video of the subject within camera view and all remaining acceleromters. We then sort those distances and obtain the rank of the matching acceleromter $R_{i}$, and thus the mean average precision (mAP) as

(13) $$\mathrm{mAP}=\frac{1}{N_{\mathrm{vid}}}\sum_{i}^{N_{\mathrm{vid}}}\frac{1}{R_{i}}\;.$$

In the second experiment, we fix $N_{\mathrm{sync}}=1$ while we vary $N_{\mathrm{vid}}$ from 2 to 10, which simulates a condition where a total of $N_{\mathrm{vid}}$ people are in front of the camera, but only one subject is being monitored with the accelerometer and the rest are guests. We measure again the mAP, as per Equation (13), to estimate the performances in retrieving the accelerometer of the monitored participant against the guests.

In the last experiment, we keep $N_{\mathrm{vid}}=N_{\mathrm{acc}}=N_{\mathrm{sync}}$ and we change their value from 2 to 10. This simulates a condition where all the participants are in front of the camera simultaneously and each is wearing an accelerometer. For each value of N=2,…,10 we measure the N×N distance matrix between all the videos and the accelerations as $D=d(V_{i},A_{j})$ for all i,j=2,…,10.

To each video clip, we then assign an acceleration sequence based on the minimum distance,

(14) $$\left(V_{i},A_{k}\right),\;k_{i}=\underset{j}{\arg\;\min}\left\{D_{i,j}\right\}\;,$$

and compute the assignment accuracy as

(15) $$\mathrm{Accuracy}=\frac{1}{N_{\mathrm{vid}}}\sum_{i=1}^{N_{\mathrm{vid}}}\left\{\delta_{i,k_{i}}\right\}\;,$$

where $\delta_{i,j}$ is the Kronecker delta.

**Figure 8:**
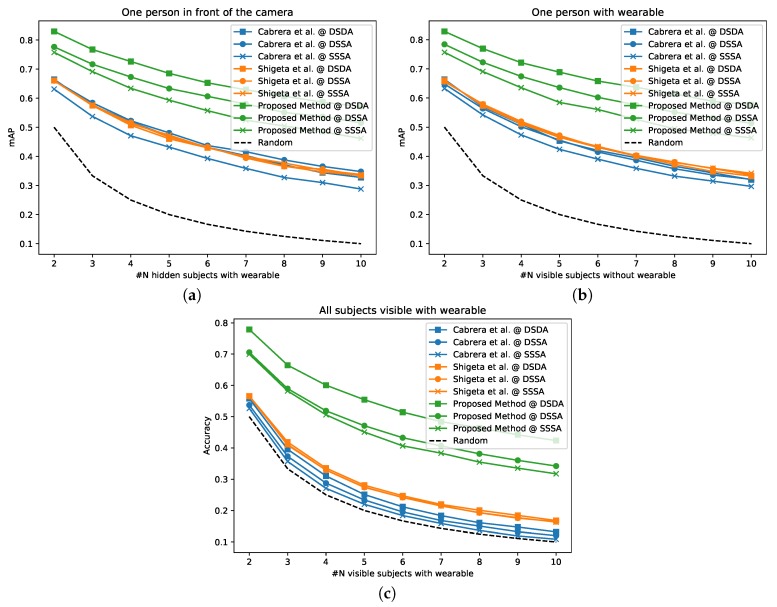
Results showing mAP and assignment accuracy for different experiments varying the number of people.

The results of these experiments are presented in Figure 8, which shows that our method outperforms the baselines for every case studied, in spite of a degradation of performances when the number of people increases from 2 to 10. Despite Cabrera et al. [42] being explicitly designed to deal with mingling events and crowded scenes, when their algorithm is applied to the short video clips, their performances drastically drop, with results that are almost on par with random guesses in the hardest scenario (i.e., DSSA, SSSA in Figure 8c).

### 4.4. Variable Clip Length

One of the novelties of our method is its ability to cope with very short clips when matching video and acceleration streams. While we are able to accurately assign each video stream to the correct accelerometer with just ≈3 s of data, the simultaneous lack of motion in both modalities can hinder a correct association. This effect can easily be mitigated by increasing the observation time for each stream. We considered all the three subjects from the validation split (8, 9 and 10) and simulated an experiment where they all appear in front of the camera while wearing the accelerometer. The assignment accuracy was computed again as in the previous section, based on the minimum distance between each of the modalities. We then increased the number of clips observed for each subject and considered the average distance to study the behaviour of the assignment accuracy for a varying time interval.

**Figure 9:**
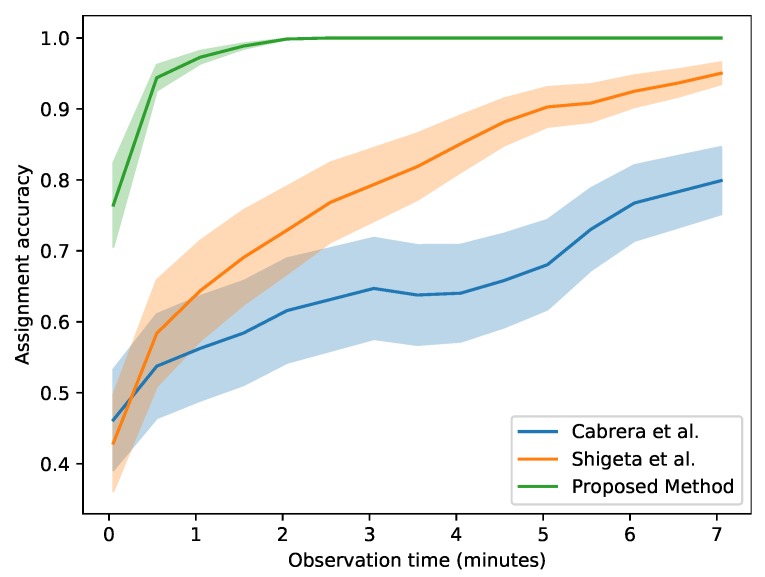
Assignment accuracy for different lengths of observation time. The area coloured in shade represents the variance for each plot.

Figure 9 illustrates the performances of each method for a variable observation time. The plots show that the baseline methods improve drastically with longer observation times, while our proposed algorithm hugely outperforms them by saturating to almost 100% assignment accuracy after only 2 min of observation time.

## 5. Conclusions

Novel technologies like IoT and AAL are becoming increasingly more popular and can potentially produce a change in the current paradigm of healthcare. An important aspect that needs to be carefully considered while working with these technologies is privacy, and video silhouettes have already shown a great potential in this regard, allowing for digital health monitoring while overcoming the ethical restrictions imposed by the use of traditional cameras. In spite of their compatibility with privacy concerns, silhouettes anonymity is a double-edged sword that both prevents identification of the household and hinders the ability to identify and track the progress of monitored subjects amongst others.

In this paper, we developed a novel deep learning algorithm that allows the identification and tracking of the monitored individuals thanks to the matching of video sequences from silhouettes with the acceleration from a wearable device carried by the subject. Differently to previous works, our algorithm is able to work also in the presence of guests as it requires only the monitored subject to be wearing the accelerometer. Moreover, our algorithm outperforms previous works by enabling the matching of video and acceleration clips of very short durations (≈3 s), making it highly suitable for short and clinically relevant movements like the transition from sitting to standing. We demonstrate the validity of our results in a series of experiments and ablation studies, presenting an average auROC of 76.3% and an assignment accuracy of 77.4%. With our results, we show that a deep-learning algorithm largely outperforms traditional methods based on tailored features when tackling the Video-Acceleration Matching problem.

## Figures and Tables

**Table 1:** Description of possible negative samples for the triplet learning.

	Same Activity (SA)	Different Activity (DA)	Overlapping
Same Subject (SS)	SSSA	SSDA	OVLP
Different Subject (DS)	DSSA	DSDA	

**Table 2:** Description of training strategies.

		Easy			Hard	Very Hard
		**DSDA**	**DSSA**	**SSDA**	**SSSA**	**OVLP**
Easy negatives only	Easy	50%	50%			
Mixed easy/hard negatives	Easy/Hard	25%	25%		50%	
Hard negatives only	Hard				100%	
Mixed hard/very-hard negatives	Hard/VeryH				50%	50%
Very-hard negatives only	VeryH					100%
Mixed easy, hard, very-hard negatives	All	11%	11%	11%	33%	33%

**Table 3:** Results of auROC for *fully-conv*.

	Standard Triplet Loss					Reciprocal Triplet Loss				
	***DSDA***	***DSSA***	***SSSA***	***OVLP***	***AVG***	***DSDA***	***DSSA***	***SSSA***	***OVLP***	***AVG***
**Easy**	65.3	60.3	57.1	55.6	**59.6**	84.4	75.5	69.1	62.7	72.9
**Easy/Hard**	61.2	58.6	56.9	55.4	58.0	82.9	76.8	73.6	72.0	**76.3**
**Hard**	59.2	58.5	57.2	55.7	57.6	79.9	79.2	74.8	70.5	76.1
**Hard/VeryH**	59.7	59.5	57.3	55.5	58.0	79.7	78.1	75.0	72.1	76.2
**VeryH**	59.7	59.2	58.1	56.7	58.4	78.7	78.1	74.1	72.4	75.8
**All**	60.9	58.6	56.8	55.0	57.8	81.4	77.8	73.5	69.8	75.6

**Table 4:** Results of auROC for Long Short-Term Memory *(LSTM)+TP*.

	Standard Triplet Loss					Reciprocal Triplet Loss				
	***DSDA***	***DSSA***	***SSSA***	***OVLP***	***AVG***	***DSDA***	***DSSA***	***SSSA***	***OVLP***	***AVG***
**Easy**	62.5	58.6	55.4	52.5	57.3	69.3	60.7	57.8	51.1	59.7
**Easy/Hard**	66.1	61.5	59.7	57.2	61.1	70.3	65.1	59.3	53.2	62.0
**Hard**	64.3	62.2	59.4	57.7	60.9	67.8	65.1	58.7	52.4	61.0
**Hard/VeryH**	64.0	62.3	61.0	59.4	**61.7**	67.8	65.0	57.1	53.6	60.9
**VeryH**	61.4	60.6	59.3	57.3	59.7	68.1	65.9	60.3	54.2	**62.1**
**All**	61.7	59.4	57.5	55.7	58.6	69.6	64.1	58.2	53.1	61.2

**Table 5:** Results of auROC for *LSTM*.

	Standard Triplet Loss					Reciprocal Triplet Loss				
	***DSDA***	***DSSA***	***SSSA***	***OVLP***	***AVG***	***DSDA***	***DSSA***	***SSSA***	***OVLP***	***AVG***
**Easy**	66.7	59.7	57.5	54.6	59.7	70.1	60.5	55.9	50.9	59.3
**Easy/Hard**	63.8	62.2	59.4	58.7	**61.0**	70.2	63.2	59.0	53.3	61.4
**Hard**	60.3	60.1	58.9	58.3	59.4	74.1	73.8	68.0	64.9	70.2
**Hard/VeryH**	57.1	57.0	57.0	56.2	56.8	74.7	72.6	69.6	67.5	**71.1**
**VeryH**	56.3	56.1	55.4	55.2	55.8	74.1	72.2	69.4	67.7	70.8
**All**	55.1	55.0	53.9	53.7	54.4	72.8	69.0	64.9	62.5	67.3

## References

[1] Maskeliūnas R., Damaševičius R., Segal S. (2019). A Review of Internet of Things Technologies for Ambient Assisted Living Environments. Future Internet.

[2] Sathyanarayana S., Satzoda R.K., Sathyanarayana S., Thambipillai S. (2018). Vision-based patient monitoring: A comprehensive review of algorithms and technologies. J. Ambient Intell. Humaniz. Comput..

[3] Zagler W., Panek P., Rauhala M. (2008). Ambient Assisted Living Systems—The Conflicts between Technology, Acceptance, Ethics and Privacy. Assisted Living Systems—Models, Architectures and Engineering Approaches.

[4] Ziefle M., Rocker C., Holzinger A. Medical Technology in Smart Homes: Exploring the User’s Perspective on Privacy, Intimacy and Trust. Proceedings of the IEEE Annual Computer Software and Applications Conference Workshops.

[5] Birchley G., Huxtable R., Murtagh M., ter Meulen R., Flach P., Gooberman-Hill R. (2017). Smart homes, private homes? An empirical study of technology researchers’ perceptions of ethical issues in developing smart-home health technologies. BMC Med. Ethics.

[6] Hall J., Hannuna S., Camplani M., Mirmehdi M., Damen D., Burghardt T., Tao L., Paiement A., Craddock I. Designing a Video Monitoring System for AAL applications: The SPHERE Case Study. Proceedings of the IET International Conference on Technologies for Active and Assisted Living.

[7] Chaaraoui A.A., Climent-Pérez P., Flórez-Revuelta F. (2012). A review on vision techniques applied to Human Behaviour Analysis for Ambient-Assisted Living. Expert Syst. Appl..

[8] Masullo A., Burghardt T., Damen D., Hannuna S., Ponce-Lopez V., Mirmehdi M. CaloriNet: From silhouettes to calorie estimation in private environments. Proceedings of the British Machine Vision Conference.

[9] Masullo A., Burghardt T., Perrett T., Damen D., Mirmehdi M. (2019). Sit-to-Stand Analysis in the Wild Using Silhouettes for Longitudinal Health Monitoring. Image Analysis and Recognition.

[10] Akagündüz E., Aslan M., Şengür A., Wang H., İnce M.C. (2017). Silhouette Orientation Volumes for Efficient Fall Detection in Depth Videos. IEEE J. Biomed. Health Inform..

[11] Nieto-Hidalgo M., Ferrández-Pastor F.J., Valdivieso-Sarabia R.J., Mora-Pascual J., García-Chamizo J.M. (2016). A vision based proposal for classification of normal and abnormal gait using RGB camera. J. Biomed. Inform..

[12] Colantonio S., Coppini G., Giorgi D., Morales M.A., Pascali M.A., Leo M., Farinella G.M. (2018). Chapter 6—Computer Vision for Ambient Assisted Living: Monitoring Systems for Personalized Healthcare and Wellness That Are Robust in the Real World and Accepted by Users, Carers, and Society. Computer Vision for Assistive Healthcare.

[13] Zhu N., Diethe T., Camplani M., Tao L., Burrows A., Twomey N., Kaleshi D., Mirmehdi M., Flach P., Craddock I. (2015). Bridging e-Health and the Internet of Things: The SPHERE Project. IEEE Intell. Syst..

[14] Grant S., Blom A.W., Whitehouse M.R., Craddock I., Judge A., Tonkin E.L., Gooberman-Hill R. (2018). Using home sensing technology to assess outcome and recovery after hip and knee replacement in the UK: The HEmiSPHERE study protocol. BMJ Open.

[15] Masullo A., Burghardt T., Damen D., Perrett T., Mirmehdi M. Who Goes There? Exploiting Silhouettes and Wearable Signals for Subject Identification in Multi-Person Environments. Proceedings of the IEEE International Conference on Computer Vision Workshops.

[16] Tao L. (2016). SPHERE-Calorie.

[17] Tao L., Burghardt T., Mirmehdi M., Damen D., Cooper A., Hannuna S., Camplani M., Paiement A., Craddock I. (2017). Calorie Counter: RGB-Depth Visual Estimation of Energy Expenditure at Home.

[18] Yao Z., Wu X., Xiong Z., Ma Y. (2019). A Dynamic Part-Attention Model for Person Re-Identification. Sensors.

[19] Gohar I., Riaz Q., Shahzad M., Ul Hasnain Hashmi M.Z., Tahir H., Ehsan Ul Haq M. (2020). Person Re-Identification Using Deep Modeling of Temporally Correlated Inertial Motion Patterns. Sensors.

[20] Zeng Z., Wang Z., Wang Z., Zheng Y., Chuang Y.Y., Satoh S. (2020). Illumination-adaptive person re-identification. IEEE Trans. Multimed..

[21] Bedagkar-Gala A., Shah S.K. (2014). A survey of approaches and trends in person re-identification. Image Vis. Comput..

[22] Wu D., Zheng S.J., Zhang X.P., Yuan C.A., Cheng F., Zhao Y., Lin Y.J., Zhao Z.Q., Jiang Y.L., Huang D.S. (2019). Deep learning-based methods for person re-identification: A comprehensive review. Neurocomputing.

[23] Layne R., Hannuna S., Camplani M., Hall J., Hospedales T.M., Xiang T., Mirmehdi M., Damen D. A Dataset for Persistent Multi-target Multi-camera Tracking in RGB-D. Proceedings of the IEEE Conference on Computer Vision and Pattern Recognition Workshops.

[24] Munaro M., Fossati A., Basso A., Menegatti E., Van Gool L. (2014). One-Shot Person Re-identification with a Consumer Depth Camera. Person Re-Identification.

[25] Nambiar A., Bernardino A., Nascimento J.C. (2019). Gait-based Person Re-identification. ACM Comput. Surv..

[26] Wang L., Tan T., Ning H., Hu W. (2003). Silhouette analysis-based gait recognition for human identification. IEEE Trans. Pattern Anal. Mach. Intell..

[27] Gou M., Zhang X., Rates-Borras A., Asghari-Esfeden S., Sznaier M., Camps O. (2016). Person Re-identification in Appearance Impaired Scenarios. arXiv.

[28] Zhang P., Wu Q., Xu J., Zhang J. Long-Term Person Re-identification Using True Motion from Videos. Proceedings of the IEEE Winter Conference on Applications of Computer Vision.

[29] Bredin H., Chollet G. (2007). Audiovisual Speech Synchrony Measure: Application to Biometrics. EURASIP J. Adv. Signal Process..

[30] Arandjelovic R., Zisserman A. Look, Listen and Learn. Proceedings of the IEEE International Conference on Computer Vision.

[31] Roth J., Chaudhuri S., Klejch O., Marvin R., Gallagher A., Kaver L., Ramaswamy S., Stopczynski A., Schmid C., Xi Z. AVA-ActiveSpeaker: An Audio-Visual Dataset for Active Speaker Detection. Proceedings of the ICASSP 2020—2020 IEEE International Conference on Acoustics, Speech and Signal Processing (ICASSP).

[32] Chung J.S., Zisserman A. (2018). Learning to lip read words by watching videos. Comput. Vis. Image Underst..

[33] Korbar B., Tran D., Torresani L. Cooperative Learning of Audio and Video Models from Self-Supervised Synchronization. Proceedings of the 2018 Conference on Neural Information Processing Systems.

[34] Teixeira T., Jung D., Savvides A. Tasking networked CCTV cameras and mobile phones to identify and localize multiple people. Proceedings of the ACM International Conference on Ubiquitous Computing.

[35] Jiang W., Yin Z. (2017). Combining passive visual cameras and active IMU sensors for persistent pedestrian tracking. J. Vis. Commun. Image Represent..

[36] Henschel R., Marcard T.V., Rosenhahn B. Simultaneous Identification and Tracking of Multiple People Using Video and IMUs. Proceedings of the IEEE Conference on Computer Vision and Pattern Recognition Workshops.

[37] Jimenez A., Seco F., Prieto C., Guevara J. A comparison of Pedestrian Dead-Reckoning algorithms using a low-cost MEMS IMU. Proceedings of the IEEE International Symposium on Intelligent Signal Processing.

[38] Shigeta O., Kagami S., Hashimoto K. Identifying a moving object with an accelerometer in a camera view. Proceedings of the IEEE/RSJ International Conference on Intelligent Robots and Systems.

[39] Rofouei M., Wilson A., Brush A., Tansley S. Your phone or mine?: Fusing body, touch and device sensing for multi-user device-display interaction. Proceedings of the ACM Annual Conference on Human Factors in Computing Systems.

[40] Wilson A.D., Benko H. Crossmotion: Fusing device and image motion for user identification, tracking and device association. Proceedings of the International Conference on Multimodal Interaction.

[41] Cabrera-Quiros L., Hung H. Who is where? Matching People in Video to Wearable Acceleration During Crowded Mingling Events. Proceedings of the ACM on Multimedia Conference.

[42] Cabrera-Quiros L., Hung H. (2019). A Hierarchical Approach for Associating Body-Worn Sensors to Video Regions in Crowded Mingling Scenarios. IEEE Trans. Multimed..

[43] OpenNI. https://structure.io/openni.

[44] Schroff F., Kalenichenko D., Philbin J. FaceNet: A unified embedding for face recognition and clustering. Proceedings of the IEEE Conference on Computer Vision and Pattern Recognition.

[45] Hinton G., Deng L., Yu D., Dahl G.E., Mohamed A.-R., Jaitly N., Senior A., Vanhoucke V., Nguyen P., Sainath T.N. (2012). Deep Neural Networks for Acoustic Modeling in Speech Recognition: The Shared Views of Four Research Groups. IEEE Signal Process. Mag..

[46] Bredin H. TristouNet: Triplet loss for speaker turn embedding. Proceedings of the 2017 IEEE International Conference on Acoustics, Speech and Signal Processing.

[47] Torfi A., Dawson J., Nasrabadi N.M. Text-Independent Speaker Verification Using 3D Convolutional Neural Networks. Proceedings of the IEEE International Conference on Multimedia and Expo.

[48] Chen C., Jafari R., Kehtarnavaz N. (2017). A survey of depth and inertial sensor fusion for human action recognition. Multimed. Tools Appl..

[49] Lagadec R., Pelloni D., Weiss D. A 2-channel, 16-bit digital sampling frequency converter for professional digital audio. Proceedings of the IEEE International Conference on Acoustics, Speech, and Signal Processing.

[50] Cao Z., Simon T., Wei S.E., Sheikh Y. (2018). Realtime Multi-Person 2D Pose Estimation using Part Affinity Fields. arXiv.

[51] Cabrera-Quiros L., Demetriou A., Gedik E., van der Meij L., Hung H. (2018). The MatchNMingle dataset: A novel multi-sensor resource for the analysis of social interactions and group dynamics in-the-wild during free-standing conversations and speed dates. IEEE Trans. Affect. Comput..

[52] Kingma D.P., Ba J. (2014). Adam: A method for stochastic optimization. arXiv.

